# A Word with the Cook

**Published:** 1888-01-28

**Authors:** 


### THE DOCTOR'S PULPIT.

### A WORD WITH THE COOK.

BUT who is the cook? That is the first point to settle. Is she the mistress of the house, with only one helper in the shape of a cheap and inefficient maid-of-all-work? Or is she a stout and important person of forty, with a competent kitchen staff and unlimited command of the purse? Or is she a *Frenchman* with a full-moon face, a paper cap, and £300 a-year?

The preacher professes his entire inability to say a single word on the art and mystery of cooking at which the *chef* would not shrug his shoulders with truly sublime and truly French pity. Even the stout person in the kitchen is sufficiently alarming to a man of moderate courage; and he feels a decided shrinking from the thought of offering any, possibly unwelcome, instruction to her. But the lady upstairs, the clergyman's young wife, or the young doctor's new bride, he does not feel nearly so much afraid of, and to them, therefore, first and chiefly, this brief contribution is offered.

You, who have been brought up tenderly, and know something of the world of cooks as well as of domestic cares, are surely not ashamed of cooking! If you are, and your husband's income is only of moderate dimensions, your friends may bid adieu to any visions of prosperity for your family. The management of the food is the key to the family expenditure as a rule, and expenditure means prosperity or failure. Mr. Micawber was never more correct in his life than when he enunciated that fundamental axiom of political, social, domestic, and every other kind of economy—"Income, £20: expenditure, £19 19*s.* 6*d.*—happiness! Income, £20: expenditure, £20 0*s.* 6*d.*—misery." If any mistress of a moderate income is ashamed of cooking or of knowing all about it, her husband will probably have cause to be ashamed both of her and of himself before many years shall have passed away.

Few *men* will question our dictum when we say that the gentler sex do not attach sufficient importance to the daily cooking question. Ladies do not relish their food with the same keenness as men, and they require less of it. Living indoors the greater part of the day they acquire the habit of feeding sparely, of being content with little snatches now and again, of tea-drinking, cake-eating, and other such trifling with important bodily functions. That is both foolish and injurious. The occupant of the pulpit, with all his sincere appreciation of the gentler and more spiritual qualities of the sex, cannot acquit them of inclining to the invertebrate condition, and this is especially the case with many in relation cookery.

Men, on the other hand, have very decided ideas about food and feeding. They may submit to a good deal of imperfection for the sake of peace, and because they cannot forget the days when dear Edith was the sweetest and most delightful of all human creatures. But though they say little or nothing it is not because they do not think. If a man has spoken once or twice he will rather endure than raise a domestic storm. But if his clerk or his tailor served him as inefficiently in their line of things as his wife does in the management of the table, he would change either of them in a week. He cannot, however, change his wife; and perhaps he would not if he could, knowing that there is such a possibility as jumping out of the frying-pan and finding himself in a still hotter place.

Seriously, men do regard the table and its management as questions of practical moment; and the doctor feels bound, as a man of science, to confirm their view of the case. That civilisation cannot be called uniformly advanced in which the cooking is in a coarse, backward, and unappetising condition. If the more amiable sex rail at us, and call us gourmands and other opprobrious names, we must be content to admit the impeachment in the sense that we cannot regard cookery as other than important: and we must ask them to take us and deal with us as they find us.

What is the first requisite of an outspread table? Is it not this—that it shall look tempting to hunger? What is the second? It is that the food shall be as pleasant to the taste as to the sight, and as wholesome as it is pleasant. The dinner is the meal of meals, and we may best enforce our special points by taking it in some detail. The ordinary every-day dinner is what we are concerned about; dinner parties we now have nothing to do with. Here is the soup; is it hot or half cold? Is it clear and giving off an odorous fragrance, or is it covered with a greasy film? Nothing, we would suppose, should be easier than to make soup wholesome, palatable, pleasant to the sight, and stimulating to the appetite. There is no necessity for hurrying it in the making; no need to run the risks of the chapter of accidents. It can be made, clarified, and seasoned the day before, and with a little foresight can always be depended upon to come to the table in a perfect condition. Even Mary Jane in the kitchen can be absolutely prevented from bungling the soup by sober and decided forethought on the mistress's part.

The fish we admit is a little more difficult to manage, and melted butter or other accompaniment will go wrong on occasions. But where there is only one servant, and soup is available, the fish may often be omitted. The beef, however, or some other animal food, is absolutely indispensable every day; and the potatoes come under the same category. It is in the management of the meat and potatoes that commonplace people fail. Anybody who can make a fire can roast a sirloin; but the sirloin will be cold beef the next day, and if any remains for the third, it may be hash or resurrection pie, or some other compound, which, if not made with great taste and skill is not fit for anybody but a ploughman either to look at or to eat. It is here where the really clever woman shines, and it is here where saving can be effected. If the leg of mutton or sirloin can always be made presentable and palatable for three instead of two days' dinners, a very considerable difference will be made in the year's expenditure. South country housekeepers often forget how many adjuncts are possible on the cold beef or hash days. Suet dumplings (now called puddings) are excellent additions when properly made, both for men, women, and children; and with a nice gravy they are much relished and make the hash quite endurable. Similarly, batter puddings, baked or boiled, are much liked by men and children, and may be eaten with meat in a variety of ways. There is no doubt that middle-class people of sedentary habits eat too much meat; and this is not due to the fact that they like it most, but that it gives less trouble to the cook than the particular puddings and dumplings which can be eaten with it. There is very great scope for improvement in this department of dinner.

The potato is a most illused vegetable. The way in which mediocre cooks spoil it five days out of six is a disgrace to cookery. What can possibly be nicer than a well-cooked potato, be it steamed, or boiled, or baked, or fried, or made into chips, or even mashed? It is a vegetable capable of an infinite variety of manipulation, whereas the rule in middle-class houses is always to boil it, and to boil it badly. The least that a potato has a right to expect at the cook's hands is that it shall be steamed and brought to the table hot, dry, and floury. Badly-cooked potatoes will spoil the best dinner and the best temper in the world.

Puddings constitute a standing dish among plain-living people, and their larger use is to be encouraged. They not only save the meat, but being bulky, as they ought to be, they satisfy the appetite without putting any undue strain upon the digestion. Heavy puddings are a great mistake. "Roley-poleys," and other such compounds, are very wholesome and digestible when well made, but as often seen they are as difficult of digestion as cannon balls. There can be no possible excuse for want of variety in puddings—their name is legion; and every skilful housewife should begin at the beginning of the "Pudding Chapter" in the cookery book and go religiously through to the end, omitting only those that are indigestible or too costly for the purse.

The great wants in cookery are thought, care, decision, and labour. Before these will be given, the mistress of the house must learn to recognise the true and constant importance of the subject. On cooking health depends, and on health temper and efficiency, and on efficiency success. A year's ill-cooked dinners may make all the difference between a man's ultimately becoming a Prime Minister or a clerk in the War Department. It is said that Emerson's somewhat premature breakdown was due to his too abundant and indiscriminate consumption of every variety of cookery. If wives want cheerful and prosperous husbands, and healthy and successful children, let them above all things see to the management of the table.

